# ﻿*Nasutitermes aurantius* (Isoptera, Termitidae, Nasutitermitinae), a new nasutiform termite species from Panama and a key to soldiers of Central American *Nasutitermes* Dudley, 1890

**DOI:** 10.3897/zookeys.1256.158192

**Published:** 2025-10-17

**Authors:** Rudolf H. Scheffrahn, Menglin Wang, Thomas Bourguignon, Mauricio M. Rocha, Eliana M. Cancello, Yves Roisin, Simon Hellemans

**Affiliations:** 1 Fort Lauderdale Research and Education Center, Institute of Food and Agricultural Sciences, University of Florida, 3205 College Avenue, Davie, Florida, 33314, USA University of Florida Davie United States of America; 2 Essig Museum of Entomology, 1101 VLSB #4780, Berkeley, California, 94720, USA Essig Museum of Entomology Berkeley United States of America; 3 Okinawa Institute of Science & Technology Graduate University, 1919-1 Tancha, Onna-son, 904-0495 Okinawa, Japan Okinawa Institute of Science & Technology Graduate University Okinawa Japan; 4 Museu de Zoologia da Universidade de São Paulo, Ipiranga, São Paulo/SP, Brazil Museu de Zoologia da Universidade de São Paulo São Paulo Brazil; 5 Behavioral and Evolutionary Ecology, CP 160/12, Université Libre de Bruxelles, Av. F.D. Roosevelt 50, B - 1050 Brussels, Belgium Université Libre de Bruxelles Brussels Belgium

**Keywords:** Biodiversity, enteric valve armature, identification key, mitogenome, nasutitermitine, Neotropics, phylogenetic reconstructions, taxonomy, termites

## Abstract

*Nasutitermes aurantius***sp. nov.**, is described from Central Panama. The soldier is unique among Central American *Nasutitermes* by its small size and orange head capsule coloration. The enteric valve armature is also unique among congeners. The new species constitutes the seventh *Nasutitermes* species in the region for which the soldier is described. We provide a key to all Central American *Nasutitermes* soldiers. Our phylogenetic reconstructions indicate that *N. aurantius***sp. nov.** is more closely related to the Afrotropical *Nasutitermes lujae* than to any other Neotropical *Nasutitermes*.

## ﻿Introduction

The cosmopolitan termite genus *Nasutitermes* Dudley, 1890 is the most diverse in the World with about 250 species described (Krishna et al. 2013a). While only eight *Nasutitermes* species are previously known from Panama through Mexico, sixty-five are currently described from the Neotropics (Rocha et al. 2024; Scheffrahn et al. 2024). One invasive species, *N. corniger* (Motschulsky, 1855), remains established in southeastern Florida after an attempted eradication program (Scheffrahn et al. 2014). It has also been introduced to Abaco Island, The Bahamas (Scheffrahn et al. 2016). *Nasutitermes corniger*, no doubt, was transported to non-native localities via maritime movement of infested vessels (Scheffrahn et al. 2005).

Currently, Central America includes *N. corniger*, *N. callimorphus* Mathews, 1977, *N. ephratae* (Holmgren, 1910), *N. glabritergus* Snyder & Emerson, 1949, *N. guayanae* (Holmgren, 1910), and *N. nigriceps* (Haldeman, 1854). The taxonomic history of these species is summarized in Krishna et al. (2013b). Herein, we describe *Nasutitermes aurantius* sp. nov. from Panama and provide a key to the described *Nasutitermes* soldiers of Central America.

## ﻿Material and methods

**Figure 1.**
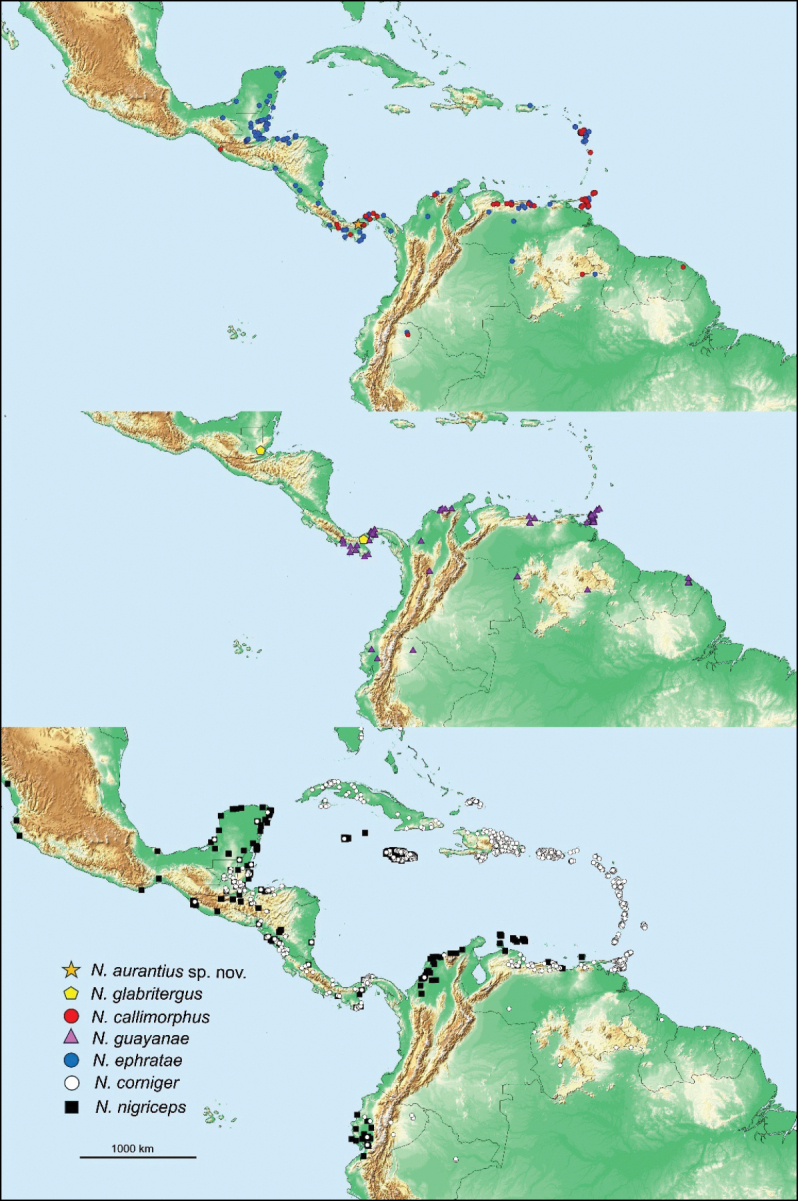
*Nasutitermes* species of Panama, Central America, and the Neotropical extent of their range.

Photomicrographs were taken as multilayer montages using a Leica M205C stereomicroscope controlled by Leica Application Suite v. 3 software. Preserved specimens were taken from 85% ethanol and suspended in a pool of Purell Hand Sanitizer to position the specimens on a transparent Petri dish background. The worker’s enteric valve armature (EVA) was prepared by removing the entire P2 section in ethanol. Food particles were expelled from the P2 tube by pressure manipulation. The tube was quickly submerged in a droplet of PVA medium (BioQuip Products Inc.), which by further manipulation, eased muscle detachment. The remaining EVA cuticle was longitudinally cut, splayed open, and mounted on a microscope slide using the PVA medium. The EVA preparation was photographed with a Leica CTR 5500 compound microscope using the same montage software. Photographs of other Central American *Nasutitermes* species were obtained from specimens in the University of Florida Termite Collection (UFTC, Scheffrahn 2019). The distribution map (Fig. 1) was produced using ArcGIS Pro Intelligence 3.0 software (Redlands, Calif.). Measurements were made following the parameters of Roonwal (1970).

### ﻿Sequencing of samples

Here, we report the sequencing of 17 new samples. Mitogenomes were sequenced according to two strategies: (1) The whole mitogenomes were amplified in two long-range PCR reactions using the TaKaRa LA Taq polymerase, primer sets and PCR conditions described in Bourguignon et al. (2016). Libraries were sequenced on the Illumina MiSeq2000 platform; and (2) Alternately, whole genomes were paired-end sequenced at low coverage with the NovaSeq 6000 Illumina platform at a read length of 150 bp. In both cases, whole genomic DNA was extracted using the DNeasy Blood & Tissue extraction kit (Qiagen), and libraries were prepared using the NEBNext® UltraTM II FS DNA Library Preparation Kit (New England Biolabs) and the Unique Dual Indexing Kit (New England Biolabs). The library preparation generally followed the manufacturer’s guidelines, but reagents were reduced to one-fifteenth of recommended volumes, and the enzymatic fragmentation step was set to a maximum of five minutes to avoid over-fragmentation. Libraries were pooled in equimolar concentration.

Raw reads were quality-trimmed using fastp v. 0.20.1 (Chen et al. 2018). Trimmed reads were assembled using metaSPAdes v. 3.13 (Nurk et al. 2017), and mitogenome scaffolds were identified and annotated with MitoFinder v. 1.4 (Allio et al. 2020). Mitogenomes were deposited in GenBank under accessions: PV022512 (BA3003) from Bahamas; PV608493 (BZ186) from Belize; PV078072 (BO352) from Bolivia; PV076159 (MZUSP20115), PV076177 (MZUSP23481), PV078265 (MZUSP25733) from Brazil; PV608494 (EC471), OR684459 (EC1314) from Ecuador; PV656442 (G17-094) from French Guiana; PV656441 (GP17-03) from Guadeloupe; OR607578 (PN183), PV608495 (PN227), OR607546 (PN1315), OR607547 (PN1316) from Panama; OR607564 (PA1206) from Paraguay; and OR607541 (PU257), OR601010 (PU724) from Peru.

### ﻿Phylogenetic reconstructions

We positioned *N. aurantius* within a phylogeny reconstructed from the mitogenomes of 69 samples representative of Isoptera and Nasutitermitinae, as evidenced from the near-complete American phylogeny of Hellemans et al. (2025). The 54 samples used to anchor our reconstructions were published elsewhere (Cameron et al. 2012; Bourguignon et al. 2015, 2016, 2017; Wang et al. 2019, 2022, 2023; Hellemans et al. 2022a, b; Arora et al. 2023). The two rRNA and 22 tRNA mitochondrial genes were aligned using MAFFT v. 7.305 (Katoh and Standley 2013). The 13 protein-coding genes were translated into amino acid sequences using the transeq function of EMBOSS v. 6.6.0 (Rice et al. 2000), then aligned with MAFFT and back-translated into codon alignments using PAL2NAL v. 14 (Suyama et al. 2006). Finally, all 37 alignments were concatenated using FASconCAT-G_v. 1.04.pl (Kück and Longo 2014).

The concatenated sequence alignment was partitioned into 41 partitions: one partition with the combined rRNAs; one with the tRNAs; and each of the 13 protein-coding genes was separated into three partitions (i.e., one for each of the three codon positions). The phylogenetic tree was reconstructed with IQ-TREE v. 2.2.2.5 (Minh et al. 2020). The partitions were merged, and the top 10% were investigated using the options “-m MFP+MERGE -rcluster-max 2000 -rcluster 10” (Chernomor et al. 2016). The best-fit nucleotide substitution model was selected with the Bayesian Information Criterion using ModelFinder implemented in IQ-TREE (Kalyaanamoorthy et al. 2017). Branch supports were assessed with 1000 bootstrap replicates, both with the ultrafast algorithm (UFB) (Hoang et al. 2018) and the Shimodaira–Hasegawa approximate likelihood-ratio (SH-aLRT) test (Guindon et al. 2010).

## ﻿Taxonomy

### ﻿ Nasutitermes aurantius

Taxon classificationAnimaliaBlattodeaTermitidae

Scheffrahn sp. nov.

225650E4-D45B-59A6-B79F-E26B483F0DF4

https://zoobank.org/5D3B658F-57EC-4EC3-BEF4-C3EBB5128D25

#### Diagnosis.

The soldier of *N. aurantius* sp. nov. is unique among Central American *Nasutitermes* spp. by its small size and orange head capsule coloration. Among South American *Nasutitermes*, the *N. aurantius* soldier is closest in size and coloration to *Nasutitermes jaraguae* (Holmgren, 1910), but the former has a proportionally longer and thinner nasus. The worker EVA of *N. aurantius* is unique among Neotropical *Nasutitermes* spp. in that the distal cushion sections are sclerotized, ovoid, granulated, and spiny. The imago of *N. aurantius* is the smallest among Central American *Nasutitermes* spp. as measured by head width.

#### Description.

**Figure 2.**
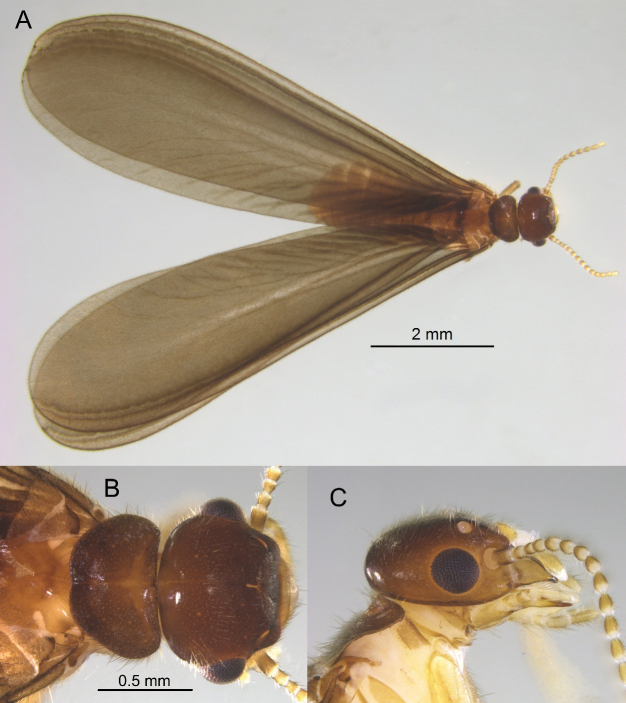
Alate of *Nasutitermes aurantius* sp. nov. (PN1317, male). A. Dorsal habitus; B. Dorsal view of head capsule and pronotum; C. Lateral view of head capsule and pronotum.

***Imago*** (Fig. 2A). Female unknown. Head capsule dark brown at vertex, gena lighter; clothed in dense setae of varying length. Fontanelle a narrow slit. Eyes nearly circular; projecting nearly half their diameter. Ocellus hyaline; more than one diameter from eye margin. Pronotum concolorous with head capsule; anterior margin straight, posterior margin slightly incised; setation as with head capsule. Antennae with 15 articles, formula 2>3<4=5, third article narrowest. Measurements (mm, *N* = 2, males): Head length to tip of labrum 1.23,1.28; head maximum width with eyes 1.21, 1.25; eye maximum diameter 0.31, 0.35; ocellus maximum diameter 0.10, 0.11; pronotum maximum length 0.56, 0.59; pronotum maximum width 0.94, 95; right forewing length from suture 9.37, 9.94; total length with wings 11.36, 11.64.

**Figure 3.**
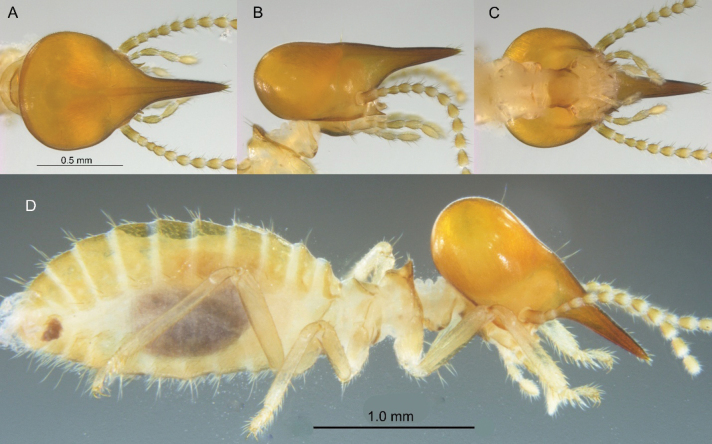
Soldier of *Nasutitermes aurantius* sp. nov. (PN1315). A. Dorsal view of head and pronotum; B. Lateral view; C. Ventral view; D. Lateral habitus.

***Soldier*** (Figs 3, 8B). Monomorphic. Head capsule orange with brown tinge, nasus darker. Head capsule subtriangulate in dorsal view; sides converging rather steeply toward nasus; slight constriction behind antennal sockets. Lateral view of head capsule with slight convexity above and behind antennal sockets. Vertex with four long setae near summit of convexity and two long setae near posterior third of vertex. Nasus narrowly conical with conspicuous setae at tip; nasus nearly parallel with lower margin of gena. Antennae with 13 articles, formula 2<3>4<5. Mandibles with prominent points. Pronotum with shallow lobes angled 120°; margin of dorsal lobe with a few long and numerous very short setae. Tergites with several long setae on posterior margins and many short setae in interior. Postmentum occupies nearly one-fourth of total head height with shallow convexity in lateral view opposite of vertex concavity. Measurements (mm, *N* = 10, X̄, range): Head length1.53 (1.46–1.58); head length to base of nasus 0.91 (0.86–0.94); nasus length 0.62 (0.57–0.67); maximum head width 0.84 (0.77–0.89); pronotum width 0.44 (0.40–0.49); hind tibia length 0.86 (0.86–0.91).

**Figure 4.**
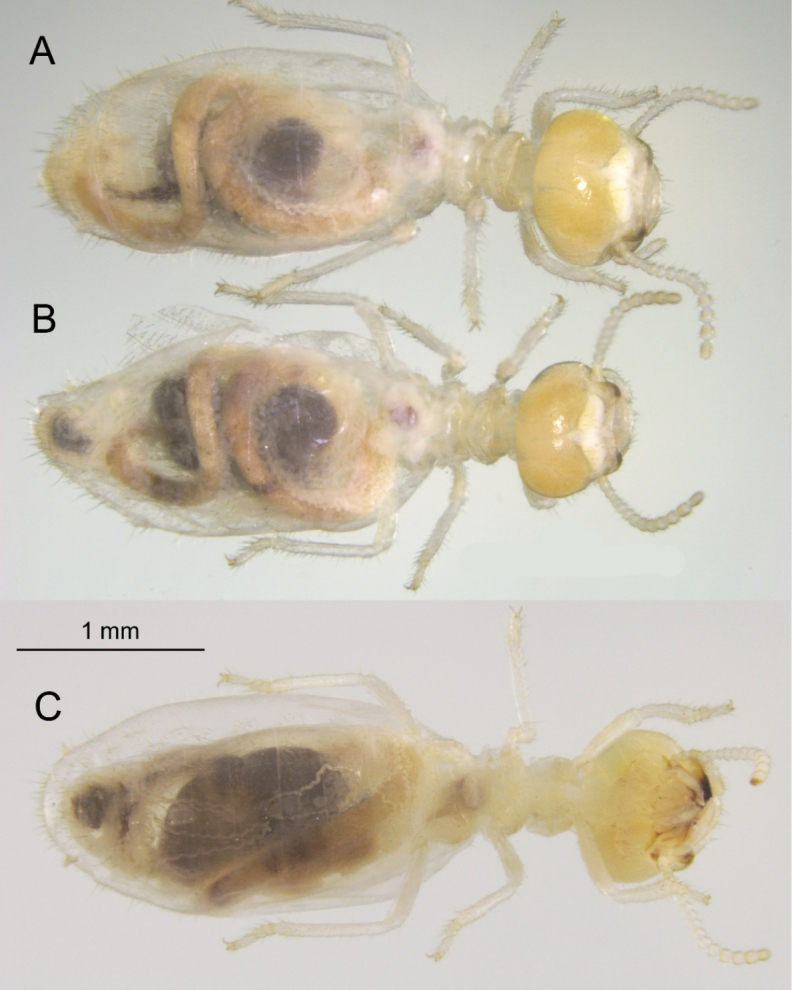
Worker of *Nasutitermes aurantius* sp. nov. (PN1315). A. Dorsal view of major worker; B. Dorsal view of minor worker; C. Ventral view of major worker.

**Figure 5.**
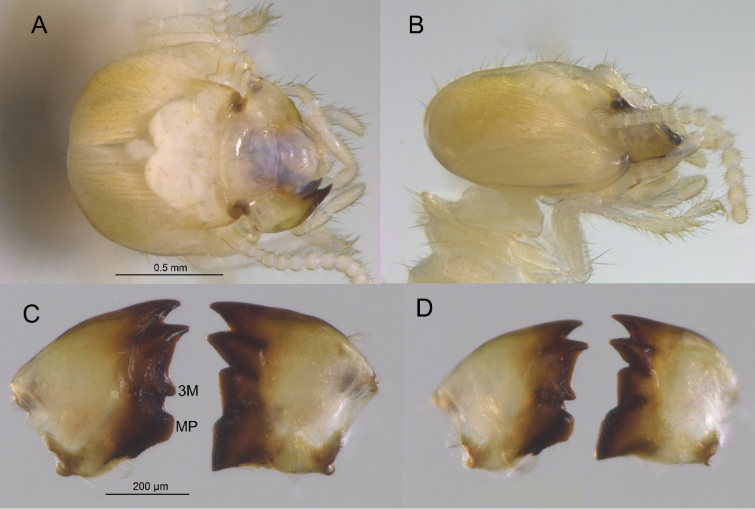
Worker of *Nasutitermes aurantius* sp. nov. (PN1315). A. Dorsal view of major worker head capsule; B. Lateral view of same; C. Major worker mandibles (3M = third marginal tooth, MP = molar prominence); D. Minor worker mandibles.

**Figure 6.**
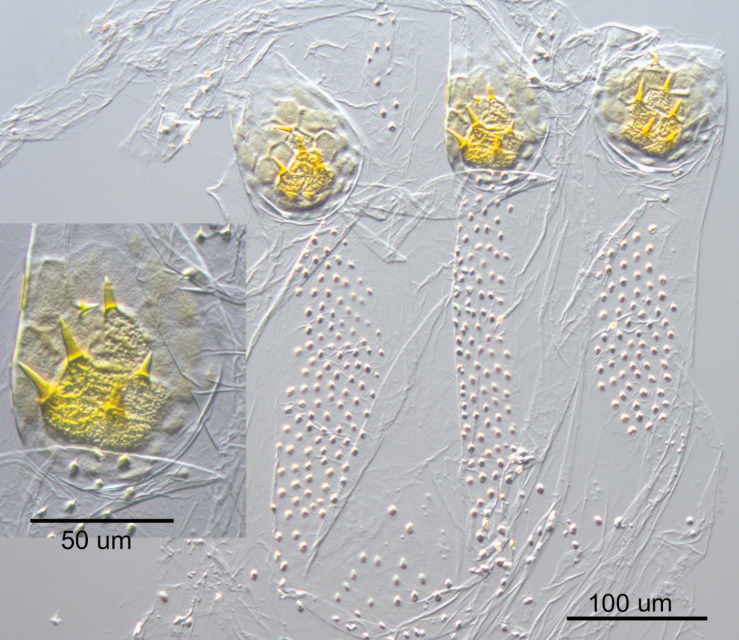
Enteric valve armature of the major worker of *Nasutitermes aurantius* sp. nov. (PN1315). Inset shows a close-up of the posterior cushion.

***Worker*** (Figs 4, 5, 6, 8B). Dimorphic. Head capsule light yellowish or light orangish; clothed with about 80 setae of varying lengths. Vertex with concavity behind postclypeus; postclypeus small, with steep posterior margin in lateral view. Fontanelle hyaline, large, ovoid; brain conspicuously white with incision carved out by fontanelle. Antennae with 14 and 13 articles for major and minor morph, respectively; antennal article formula 2>3<4=5 for both. Mandible dentition (wood feeding type) and gap between third marginal tooth and molar prominence similar for both morphs; left mandible with straight cutting edge; molar plate with ridges. Mesenteron well developed forming a complete ring from crop to P1; P1 long, tubular in dorsal and ventral views; forming an “S” from M to EVA seating visible through cuticle. Enteric valve armature composed of three cushions, each with anterior section consisting of about 80 to 100 rounded scales each with minute points facing posteriorly. Posterior sections of EVA sclerotized and ovoid; each formed with about 10–12 polygons; central 4–5 polygons granulated and each adorned with a spine; a few scattered scales between and anterior to cushions. Head width measurements (mm, *N* = 10 for each morph): major worker 1.04 X̄ (range 99–1.04); minor worker X̄ 0.87 (range 0.84–0.91).

#### Type material.

***Holotype***: Panama • Soldier; Coclé, Omar Torrijos Herrera National Park (El Cope); 8.66969°, -80.59302°; 791 m a.s.l.; 4 June 2010; R. Scheffrahn leg.; many soldiers (one labeled holotype) and workers; University of Florida Termite Collection (UFTC) no. PN1315. ***Paratypes***: Same data as holotype; two subsamples from separate colony; two male alates, many soldiers and workers, five nymphs (PN1317); many soldiers and workers, two nymphs (PN1316).

#### Etymology.

The specific name “aurantius” is from Latin “aurantium” meaning “orange”, referring to the soldier’s head capsule coloration.

#### Ecological note.

*Nasutitermes aurantius* sp. nov. foragers and two alates were collected underneath saturated decaying wood (Fig. 8). No above-ground nest structure was observed, so it is assumed that the nest is subterranean. The presence of developed alates suggests that the flight season is in early June. The type locality is classified as tropical rainforest (Köppen-Geiger) with an annual precipitation of > 250 cm.

#### Taxonomic note.

Light (1933) described two additional Central American *Nasutitermes* species from the imago caste: *N. pictus* and *N. colimae*. Both were collected simultaneously during a single dispersal flight by M. Ceballos from an “old orange tree” in Colima, Mexico. No foragers were collected. Their large size (head width 1.65 and 1.95, respectively) suggests that these two species are not *Nasutitermes* but either a *Cahuallitermes* spp. (imago unknown), a *Tenuirostritermes* spp., or an undescribed apicotermitine. Therefore, we consider *N. pictus* Light, 1933 and *N. colimae* Light, 1933 to be *nomina dubia*.

### ﻿Key to Central American *Nasutitermes* soldiers

**Table:** 

1	Head capsule yellowish or orangish (Figs 3, 7I)	**2**
—	Head capsule brown to almost black (Fig. 7A–D, H)	**3**
2	Head capsule orangish, triangulate in dorsal view (Fig. 3)	***N. aurantius* sp. nov.**
—	Head capsule yellowish, ovoid in dorsal view (Fig. 7I)	***N. glabritergus***
3	Head capsule in lateral view with four setae behind nasus and two setae near base (Fig. 7A, C, H)	**4**
—	Head capsule in lateral view with ten or more setae on dorsum (Fig. 7B, D). Nests are often very large	**6**
4	Pronotum in lateral view with no long setae on dorsal lobe, head width 0.72–0.83 mm (Fig. 7A)	***N. callimorphus***
—	Pronotum in lateral view with numerous long setae on dorsal lobe (Fig. 7E), head width variable, > 0.9 mm (Fig. 7C, H)	**5**
5	Tergite surfaces covered with barely visible microscopic setae (Fig. 7F). Alate wings golden brown; arboreal nest with smooth outer skin reminiscent of an orange peel when removed	***N. ephratae***
—	Tergite surfaces covered with clearly visible microscopic setae (Fig. 7G). Alate wings black; arboreal nest with uneven friable surface	***N. corniger***
6	Head capsule clothed with about 100 medium and long setae; setae abundant on nota (Fig. 7B)	***N. nigriceps***
—	Head capsule covered with about 30 mostly long setae; setae very sparse on nota (Fig. 7D)	***N. guayanae***

**Figure 7.**
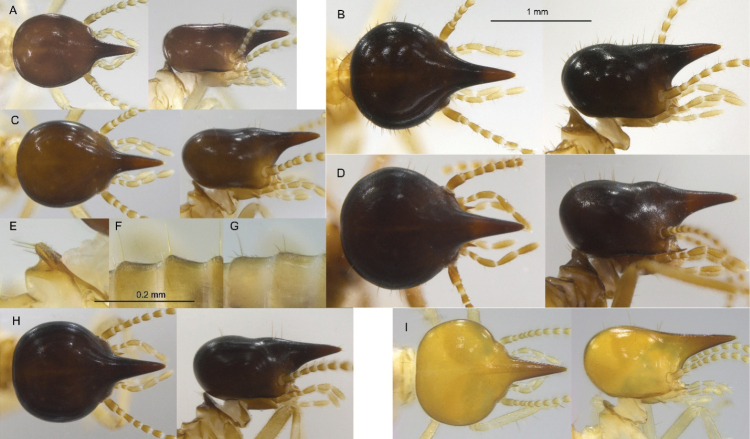
Dorsal and lateral views of soldier head capsules of Panamanian *Nasutitermes* to scale. A. *N. callimorphus*; B. *N. nigriceps*; C. *N. ephratae*; D. *N. guayanae*; E. Lateral view of the *N. ephratae* pronotum; F. Tergites of *N. corniger*; G. Tergites of *N. ephratae*; H. *N.*corniger; I. *N. glabritergus*.

**Figure 8.**
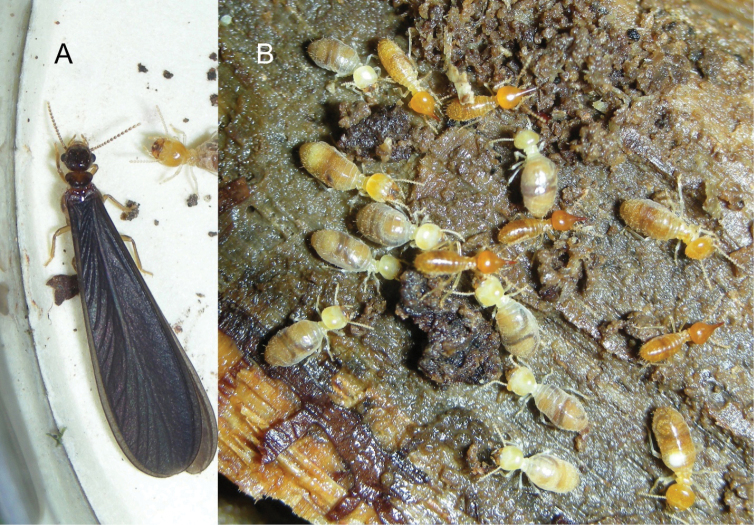
Field habitus of *Nasutitermes aurantius* sp. nov. A. Alate (PN1317); B. Foragers (PN1315; note microsporidial infection in abdomen of worker at bottom right).

## ﻿Results and discussion

**Figure 9.**
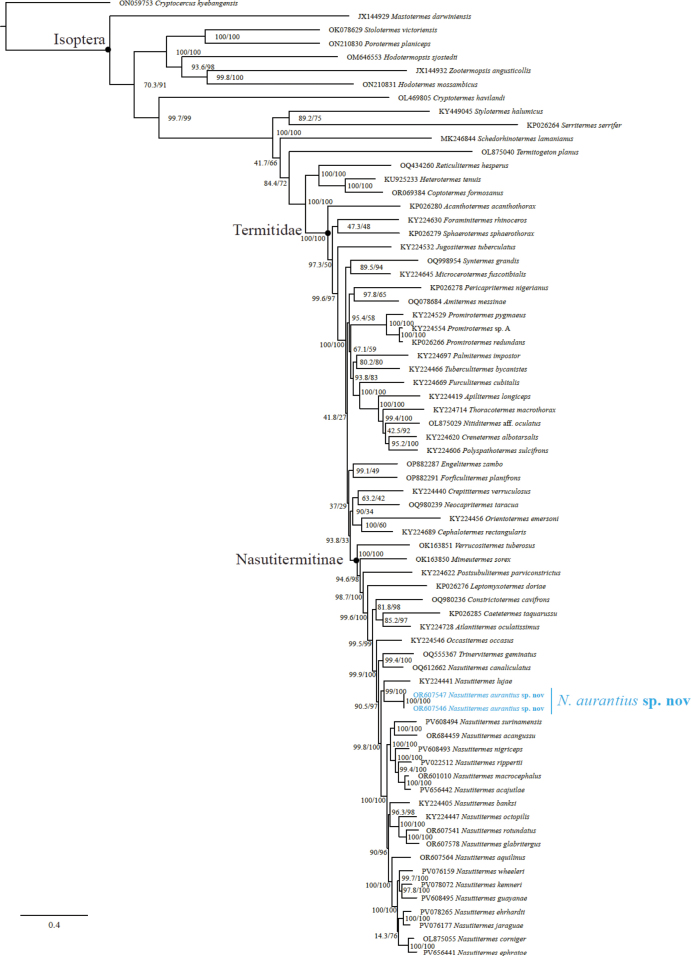
A phylogenetic tree of *N. aurantius* and other related taxa inferred from the mitogenomes. Branch supports values are the Shimodaira–Hasegawa approximate likelihood-ratio and ultrafast bootstrap (SH-aLRT/UFB).

Our phylogenetic analyses revealed that the Panamanian *N. aurantius* sp. nov. is more closely related to the Afrotropical *N. lujae* (90.61% identity) than to any other Neotropical *Nasutitermes* included herein (Fig. 9, Table 1). The sequence of *N. lujae* (KY224441) was previously published by Bourguignon et al. (2017). The taxa chosen herein for the phylogenetic tree was subset from the megaphylogeny of Hellemans et al. (2025). This paper notably included more Operational Taxonomic Units than the number of species currently described from the Neotropics. Therefore, data handling error is highly unlikely given that no other neotropical “*N. lujae*” clustered to KY224441 in the megaphylogeny by Hellemans et al (2025).

**Table 1.** Pairwise mitogenome similarities between species of Nasutitermes closely related to N. aurantius. Similarities were obtained with megaBLASTn.

SAMPLE	BA3003	BO352	BZ186	EC1314	EC471	G13-117	G17-094	G718	GP17-03	MZUSP-20115	MZUSP-23481	MZUSP-25733	NI275	PA1206	PN1315	PN1316	PN183	PN227	PU257	PU724	RDCT106
BA3003 *Nasutitermes rippertii*	100																				
BO352 *Nasutitermes kemneri*	90.345	100																			
BZ186 *Nasutitermes nigriceps*	92.768	90.639	100																		
EC1314 *Nasutitermes acangussu*	90.513	89.7	90.043	100																	
EC471 *Nasutitermes surinamensis*	90.818	89.761	90.233	90.56	100																
G13 117 *Nasutitermes banksi*	91.025	91.261	90.318	89.908	90.372	100															
G17 094 *Nasutitermes acajutlae*	92.86	90.525	93.134	90.383	90.942	91.204	100														
G718 *Nasutitermes octopilis*	90.93	90.486	89.494	90.605	89.813	90.98	90.902	100													
GP17 03 *Nasutitermes ephratae*	90.112	92.128	89.842	89.544	89.646	90.796	90.567	90.645	100												
MZUSP20115 *Nasutitermes wheeleri*	90.419	92.766	90.31	89.835	89.777	90.811	90.744	90.19	91.928	100											
MZUSP23481 *Nasutitermes jaraguae*	90.548	92.896	90.44	90.095	89.884	91.116	90.612	90.636	92.491	92.813	100										
MZUSP25733 *Nasutitermes ehrhardti*	90.481	92.983	90.101	89.68	89.771	91.07	90.642	90.588	92.376	92.687	95.164	100									
NI275 *Nasutitermes corniger*	90.632	92.652	90.132	89.845	89.834	91.225	91.168	90.104	95.419	92.477	93.008	92.887	100								
PA1206 *Nasutitermes aquilinus*	90.667	91.647	91.327	90.048	90.744	91.124	90.905	90.06	91.191	91.064	91.186	91.341	91.024	100							
PN1315 *Nasutitermes aurantius***sp. nov.**	90.347	89.886	89.927	89.441	89.78	90.4	89.986	90.269	89.465	89.771	89.993	89.964	89.977	89.954	100						
PN1316 *Nasutitermes aurantius***sp. nov.**	90.427	89.965	89.916	89.515	89.912	90.473	90.087	90.416	89.536	89.848	90.066	90.042	90.051	90.125	99.908	100					
PN183 *Nasutitermes glabritergus*	89.759	89.763	89.517	89.234	89.676	90.213	90.245	91.575	89.243	89.82	89.715	90.044	89.849	89.967	89.33	89.402	100				
PN227 *Nasutitermes guayanae*	91.266	93.732	91.686	90.312	90.303	91.786	91.373	90.849	92.738	93.258	92.839	93.19	92.899	91.104	89.623	89.775	90.266	100			
PU257 *Nasutitermes rotundatus*	90.138	90.149	89.898	89.45	89.757	90.349	90.152	92.173	89.823	90.102	90.247	90.354	89.983	90.034	89.901	89.991	93.479	90.909	100		
PU724 *Nasutitermes macrocephalus*	93.278	90.84	92.84	90.493	90.983	91.236	96.45	91.167	90.344	90.602	90.941	90.961	91.055	91.075	89.992	90.071	90.075	91.453	90.262	100	
RDCT106 *Nasutitermes lujae*	89.636	89.574	89.63	89.098	89.631	89.921	89.672	90.056	89.475	89.38	89.671	89.627	89.674	90.02	90.539	90.612	89.18	90.251	89.3	89.841	100

Our sampling includes representatives from all closely-related lineages of *Nasutitermes* according to the megaphylogeny of Hellemans et al. (2025). Furthermore, it is our “signature” to provide phylogenetic relationships within a more global framework to help non-specialists to quickly position the focal taxa in the global termite tree of life (Hellemans et al. 2022b).

The Neotropical species of the genus *Nasutitermes* are in dire need of revision. The synonymy of *Nasutitermes dasyopsis* Thorne, 1989 into *Nasutitermes nigriceps* (Haldeman, 1854) by Scheffrahn et al. (2024) leaves sixty-five valid *Nasutitermes* species in the Neotropics (Constantino 2020). Of these, thirty species may be on the precipice of taxonomic correction by scoring 4 or less out of a possible 10 on the confidence scale of Rocha et al. (2024). A low confidence score can result from lost types, descriptions of a single caste, or descriptions that are generic only, lacking detailed illustrations, biology, distribution, or genetic sequencing (Rocha et al. 2024). These species with the lowest confidence (≤ 4), originally described between 1839–1933, were plagued by poor descriptions, nomina dubia, or synonyms. Another twenty species with an intermediate confidence score (5–6) also suffered, but to a lesser extent, from mediocre descriptions, leaving only fifteen species in the upper range of confidence (≥7) (Rocha et al. 2024; Scheffrahn et al. 2024). Add to those *Nasutitermes* species that may be undescribed (UFTC database in Scheffrahn 2019), and the revised tally of Neotropical *Nasutitermes* may become profoundly amended. The recent global phylogeny of Hellemans et al. (2025) provides a good start for a systematic revision of Neotropical *Nasutitermes*.

## Supplementary Material

XML Treatment for Nasutitermes aurantius

## ﻿Additional information

### Use of AI

No use of AI was reported.

### Author ORCIDs

Rudolf H. Scheffrahn https://orcid.org/0000-0002-6191-5963

Menglin Wang https://orcid.org/0000-0003-2206-9503

Thomas Bourguignon https://orcid.org/0000-0002-4035-8977

Mauricio M. Rocha https://orcid.org/0000-0002-6568-068X

Eliana M. Cancello https://orcid.org/0000-0003-3125-6335

Yves Roisin https://orcid.org/0000-0001-6635-3552

Simon Hellemans https://orcid.org/0000-0003-1266-9134

### Data availability

All of the data that support the findings of this study are available in the main text.
